# Social determinants of serum 25-hydroxyvitamin D concentrations deficiency in older Chilean people

**DOI:** 10.1038/s41598-023-45862-1

**Published:** 2023-10-26

**Authors:** Sandra Alvear-Vega, Rodrigo Benavente-Contreras, Héctor Vargas-Garrido

**Affiliations:** 1 Faculty of Business and Economics, University of Talca, Talca, Chile; 2 Faculty of Psychology, University of Talca, Talca, Chile

**Keywords:** Biomarkers, Risk factors, Health care, Geriatrics, Health policy, Public health, Quality of life

## Abstract

Serum 25-hydroxyvitamin D concentrations deficiency is a growing health problem that affects a significant part of the world’s population, with particularly negative consequences in children and older adults. Public health has prioritized healthy aging; thus, an investigation of the social determinants related to deficient and insufficient Serum 25-hydroxyvitamin D concentrations in older adults is needed to contribute to the implementation of comprehensive social programs focused on addressing those conditions adversely affecting the health of this group. This study was conducted using a sample of older adults (age ≥ 65 years, *n* = 1283) from the National Health Survey (NHS 2016–2017). The Average Marginal Effects of the social determinants of Serum 25-hydroxyvitamin D concentrations deficiency in older adults were predicted using a probit model in which the outcome variable assumed two values (deficiency or not deficiency), taking as independent variables those reported in previous studies. The model showed an adequate goodness of fit, Count R2 = 0.65, and the independent variables explained between 11% (Cox-Snell) and 14% (Nagelkerke) of the variance of the outcome variable. The social determinants associated with a greater likelihood of Serum 25-hydroxyvitamin D concentrations deficiency are the following conditions: women, people of native origin, urban dwellers, shorter sunlight exposure, and greater geographical latitude. Implications are discussed, and limitations are considered. Promotion and prevention programs should preferentially target older adults in the southernmost regions who live in urban areas, with a special focus on women. Due to the country’s characteristics (17°–57° south latitude), it is necessary to review in future research the three zones shown in this study as relevant social determinants for the older adults living in them to generate inputs in formulating public health policies. The authorities must define the cut-off points for considering the difference between the country’s ranges of Serum 25-hydroxyvitamin D concentrations insufficiency and deficiency.

## Introduction

Serum 25-hydroxyvitamin D [25(OH)D] concentrations belong to the family of fat-soluble vitamins, although it is also proposed as a prohormone^1^. Its function is intimately related to bone health in children and adults^2–4^; it also participates in cell proliferation and differentiation as well as mental health^2^. Deficient levels of this vitamin are also associated with an increased risk of cardiovascular pathologies, cancer, diabetes, and autoimmune and infectious diseases, COVID-19, among others^5–11^. The main mechanism by which an organism obtains 25(OH)D is through exposure to sunlight, which triggers its synthesis in the skin cells thanks to the absorption of photons from UV rays^14^. It can also be obtained, although to a lesser extent, through dietary intake^4,13,14^. Thus, people with 25(OH)D deficiency mainly have less sun exposure and lower intake^15^. Some factors influencing 25(OH)D concentrations are geographical latitude, season, and ethnicity^16^.

25(OH)D deficiency is a growing health problem that affects a significant part of the world population^16–18^, with negative consequences in children, ill people, and older people^3,15,19,20^. As for the last group, the natural aging process is associated with a decrease in 25(OH)D synthesis because seven dehydrocholesterol levels in older skin are reduced^21,22^, which can be reflected in adverse health outcomes such as sarcopenia, respiratory problems, osteomalacia, bone loss, osteoporotic fractures, and muscle problems^2,6,23,24^. Along this line, public health has made older adults a priority focus of interest to generate knowledge and promote public policies for healthy aging^25^. Considering that environmental and personal factors may be involved in older adults’ lower 25(OH)D concentrations, research has begun to study the social determinants of deficient and insufficient 25(OH)D levels in this group^20,26,27^. Knowing the social conditions underlying people’s negative health results, called “the social causes of disease”^28^, can contribute to the implementation of social programs focused on addressing those adverse conditions^29^. Research on older adults has observed a lower concentration of 25(OH)D in women^20,26^, those who skip breakfast, are more sedentary, have less sun exposure, have darker skin, have disabilities, live alone, and are older^20,26,27,30–32^; especially relevant are factors such as not taking medications or vitamin supplements, smoking, the winter season, and obesity^30,32^. Likewise, protective factors negatively related to 25(OH)D deficits are intense physical activity, sleeping more than nine hours, and having more schooling and income^26,30^. On the other hand, living in rural or urban areas yields mixed results; in some cases, there is a greater deficit associated with large cities^23^, while in others, the result is the opposite^30^.

More research is also needed from Latin America and middle- and low-income countries^18,20,34^. In this regard, Chile has unique characteristics. Geographically, it is located between approximately 17° and 57° degrees south latitude, with the driest desert in the world beginning in the north (Atacama Desert) and extending to Patagonia in the south; its longitude is similar to that from southern Algeria to the north of the United Kingdom. It also possesses ethnic and lifestyle diversity and a mixture of public and private health systems that are characteristic of the Latin American reality^35^. Chilean authorities have consistently called for studies on 25(OH)D conditions in the country due to the paucity of information on specific groups. They also aim to have input to establish clear cut-off points concerning assessing 25(OH)D^36^. The present work seeks to identify the social determinants related to older adults with deficient 25(OH)D concentrations in Chile. In this context, knowledge of the social determinants can help public policymakers, health professionals, and researchers develop interventions that promote good health outcomes among the Chilean older adult population. This study considers the data provided by the National Health Survey 2016–2017^37^ and weighs a single cut-off point to define the condition of “deficiency,” based on the indications of experts from the Chilean Ministry of Health, who established the cut-off point at 20 ng/mL of 25(OH)D “to make population-based analyses that allow public policy proposals”^36^. A probit model will be run to determine the average marginal effects of each social determinant reviewed.

## Methods

### Source of information

The data used in this study are from the third version of the National Health Survey (NHS 2016–2017) commissioned by the Department of Epidemiology of the Chilean Ministry of Health^37^. Its purpose is to identify the diseases suffered by and treatments provided to men and women aged 15 years and older living in Chile. It is a cross-sectional, randomized, population-based survey stratified by cluster and applied in 15 regions of Chile in both urban and rural areas. The NHS 2016–2017 consists of three sections. The first is a questionnaire with 576 general questions (including a diagnostic interview to measure mental disorders); the second includes anthropometric measurements; and the third corresponds to laboratory tests (including measurement of 25-hydroxyvitamin concentrations). The NHS 2016–2017 database is for public use and was obtained from the Ministry of Health. All population-based surveys are conducted under Law Nº19.628 on protecting citizens' privacy, and consequently, they do not require approval by an Ethics Committee. All participants voluntarily participated. The NHS 2016–2017 is used in research as a secondary data source^27^. The total population surveyed amounted to 6,233 people. However, for the specific case of 25(OH)D, its measurement was incorporated in only two population subsamples: women of childbearing age (15 to 49 years) and the elderly (65 years and older). In this study, we will only work with the second group, composed of 1,283 older adults representative of the national level.

### Outcome variable: 25(OH)D deficiency in adults over 65 years of age

The criterion defining deficiency or not deficiency categories is the cut-off point of 20 ng/mL of 25(OH)D^13,38^. Thus, the outcome variable is dichotomous and takes the value 1 in the “25(OH)D deficiency” range (below the cut-off point) and the value 0 in the range above the cut-off point (no deficiency).

### Independent variables

The independent variables were considered according to previously reviewed literature and were present in NHS 2016–2017. They are sex, belonging or direct descent from native peoples, rural or urban areas, exposure to sunlight, and regions of the country (see Table 1). All independent variables are dichotomous, assuming the value 1 (belongs to the category) or 0 (does not belong). It should be noted that the geographic area taken as a base is the metropolitan region (located approximately between 32 and 34 degrees south latitude).

**Table 1:** Independent variables extracted from the National Health Survey (NHS) 2016–17.

Variable name	NHS 2016–2017 question	Codification
Gender	Are you male or female?	Male = 0 Female = 1
Native ethnic groups	In Chile, the law recognizes nine native populations. Do you belong to or are you a descendant of any of them?	No = 0 Yes = 1
Urban/rural residence	Which area do you live in?	Urban = 0 Rural = 1
Sunlight exposure	In the last week, how much sunlight have you been exposed to?	A lot = 0 A little = 1
Educational level	How many years of schooling do you have?	9 or more = 0 8 or below = 1
Health Insurance System	What type of health insurance do you have?	Private = 0 Public = 1
Region (15)	In which region do you live?	Others = 0 Region “X” = 1

### Plan of analysis

Data statistical analysis was performed with Stata software13. A probabilistic nonlinear model was performed with a dichotomous response (people ≥ 65 years having or not having 25(OH)D deficiency), and a regressor that refers to a probit model. Average marginal effects were calculated for each interaction, and likelihoods were predicted. The model specification is:

$$y_{i} = F + \left( \begin{gathered} \beta_{0} + \beta_{1} Sexo_{i} + \beta_{2} DescendenciapueblosO_{i} + \beta_{3} Zona_{i} + \beta_{4} LuzSolar_{i} \hfill \\ + \sum\limits_{05}^{19} {{\text{Re}} gi\mathop o{^\prime } n_{i} } \hfill \\ \end{gathered} \right) + \varepsilon_{i}$$

where *F*(.) corresponds to the cumulative distribution function assumed to be probit, which is a link between the determinants and the probability of presenting 25(OH)D deficiency; thus, the probit model to be estimated corresponds to:

1 $${\text{Probit}}(p) = \sqrt 2 erf^{ - 1} (2p - 1)$$

### Goodness of fit

The model was estimated based on 1,283 observations. The log-likelihood is -800.16, *χ*^2^ (18) = 143.24, *p* < 0.0001. The goodness of fit, Count R2 = 0.65, indicates good explanatory power of the model. The independent variables explain between 11% (Cox-Snell) and 14% (Nagelkerke) of the variance of the outcome variable.

## Results

Of the total number of older adults (*n* = 1,283) included in the present study, 59% had levels below 20.0 ng/mL of 25(OH)D, referred to as deficiency. Of the sample, 64% were women (65% had 25(OH)D deficiency), 68% reported little sunlight exposure, 20% lived in rural areas, and 7% belonged to native peoples. Moreover, 15% of older adults in the sample live in the metropolitan region (which includes the capital of Chile), 34% live in the regions located to the north, and 51% live to the south of the metropolitan region. Out of those with 25(OH)D deficiency, 16% live in the metropolitan region, 31% live in the north, and 53% live in the south of the metropolitan region. When stratified by sex and region of the country, women living in the first three regions of the north have a considerably higher 25(OH)D deficiency than men (61% vs. 25%). However, in the last three regions of the south, deficiency increases in both sexes and is close to each other (77% vs. 75%, women and men, respectively). Also, 60% of people with 25(OH)D deficiency have less than eight years of schooling or no schooling, and 59% have public health insurance (National Health Fund, called FONASA). It should be noted that according to Casen 2017, 44% of older people are within the first two quintiles, with the lowest income. Both schooling and type of health insurance were incorporated as a proxy for income level; however, their relationships with 25(OH)D deficiency were not statistically significant for a 95% confidence interval.

Next, variables with statistical significance within the model will be presented. The demographic results indicate that women are 15% more likely to have 25(OH)D deficiency than men (see Table 2). Older people with low exposure to sunlight are more likely (8%) to have 25(OH)D deficiency than those exposed to more sunlight. Also, those living in rural areas have a lower probability (-24%) of having 25(OH)D deficiency than those living in urban areas. People of native origin are 11% more likely to have 25(OH)D deficiency.

**Table 2:** Average Marginal Effects (AMEs) of the social determinants of 25(OH)D deficiency.

	Predict (25(OH)D deficiency)		
Social determinants	Codification	AMEs (dy/dx)	[95%. Conf. Interval]
Gender	Female = 1; Male = 0	0.15***	[0.089;0.208]
Native ethnic groups	Yes = 1; No = 0	0.11**	[0.005; 0.213]
Urban/rural residence	Rural = 1; Urban = 0	−0.24***	[−0.312; −0.165]
Sunlight exposure	A little = 1; A lot = 0	0.08**	[0.016; 0.140]

*p < .01; **p < .05; ***p < .001.

**Figure 1:**
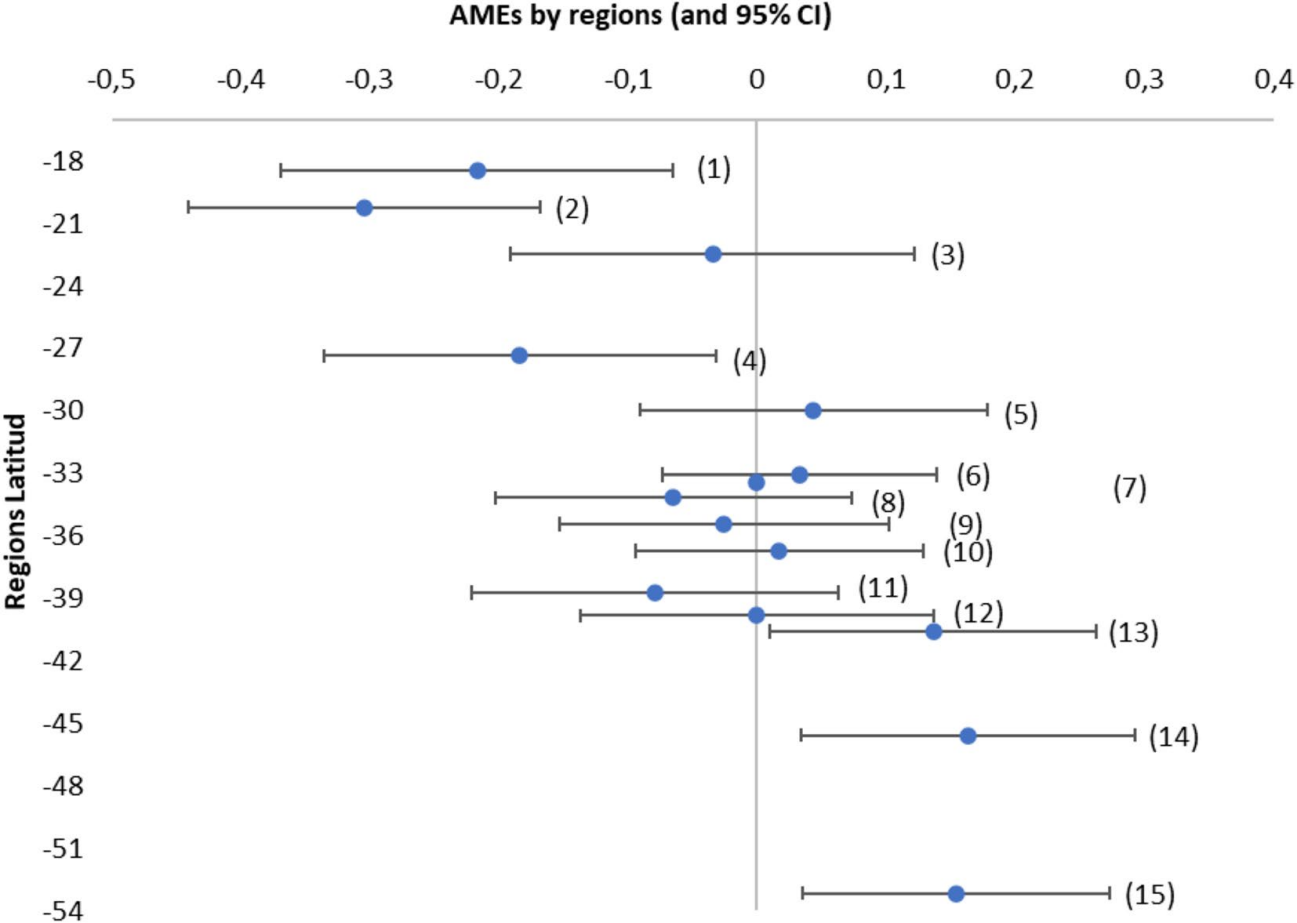
Average Marginal Effects (AMEs) of Older adults 25(OH)D deficiency, by Chilean regions. Note. Regions from north to south follow each other in order: Arica and Parinacota (1); Tarapacá (2); Antofagasta (3); Atacama (4); Coquimbo (5); Valparaíso (6); Metropolitan (7); O’Higgins (8); Maule (9); Biobío (10); Araucanía (11); Los Ríos (12); Los Lagos (13); Aysen (14), and Magallanes (15).

The regions from north to south follow each other from the lowest to greatest latitude (see Fig. 1). Older people living in the northern part of the country (regions 1, 2, and 4 closer to the Equator) are less likely to have 25(OH)D deficiency than those in the metropolitan region. At the same time, people living in the country’s extreme south (regions 13, 14, and 15, closer to Antarctica) are more likely to have 25(OH)D deficiency than people living in the metropolitan region. The other regions of the country show no significant results.

## Discussion

Using the NHS 2016–2017, our results on 25(OH)D deficiency in Chilean older adults converge with findings reported in other countries and also render important comparative information, all of which allows us to contribute to understanding this critical issue for public health that has implications for the wellbeing and quality of life of older people in the country and the world.

Regarding demographic variables, it is observed that older women have higher 25(OH)D deficiency, an issue that has been consistently reported in previous studies^20,26^. However, multiple factors could be related to these sex differences, including, for example, differences in vitamin D intake between men and women, sun exposure, skin synthesis capabilities, age, and being overweight^39^. Without a definitive answer, differences in synthesis capabilities derived from different skin pigmentation could be ruled out since Chilean women tend to have a lighter skin tone than men^40^. In addition, men tend to withdraw fewer dietary supplements than women^41^ that are fortified with vitamin D. Likewise, there is no difference in age by sex in the sample (*Males average* = 73.8 years vs. *Females average* = 74.2 years), *t*(1283) = -1.00, *p* = 0.31, and men agree the most to be exposed to more sunlight than women (*Males average* = 1.43 vs. *Females average* = 1.26), *t*(851) = 6.35, *p* < 0.001. However, a factor that might explain this difference is that Chilean women are overweight at higher levels than men^42^, a condition consistently related to lower 25(OH)D status^43,44^. In our sample, their body mass index (BMI) ratio is higher than that of men (*Males* = 0.59 vs. *Females* = 0.63), *t*(1058) = -8.05, *p* < 0.001.

The main mechanism for obtaining 25(OH)D is through exposure to sunlight^12^. Previous studies have consistently found higher 25(OH)D concentrations in the summer months^30–32^; thus, it is not surprising that in this study, those older adults who reported greater exposure to sunlight had higher 25(OH)D levels.

Latitude is also a predominant factor in 25(OH)D levels due to greater solar exposure closer to the Equator^16,32^. In our results, older adults from the northernmost regions (Arica and Parinacota, and Tarapacá), located between 17° 30′ and 21° 38′ south latitude, have the lowest likelihood of presenting 25(OH)D deficiency. The metropolitan region, which is taken as a base, does not differ from the regions located from 21° 38′ to 40° 33′ south latitude, while the areas located from that point to 56° 30′ south latitude (Los Lagos, Aysen, and Magallanes) are where older adults deliver the highest probabilities of showing 25(OH)D deficiency. Therefore, 25(OH)D in the country is associated with the geographical location north to south (north closest to the Equator). A comparison of mean 25(OH)D levels among older adults in these three geographic zones yields significant differences among them: Great North Zone (Mean = 22), from Little North to South (Mean = 19), and Austral Zone (Mean = 16), *F* (2, 1283) = 24.22, *p*s < 0.01, with insufficiencies of 37.2%, 57.8%, and 74.3%, respectively. In comparative terms, using the same cut-off point, the rates of deficiency move in different ranges in the world, for example, from 21.6% in Ecuador^34^ and 23.5% in Brazil^20^ to 62.1% in Korea^26^ and 67.2% in Portugal^30^. Thus, using the only cut-off point suggested by the authorities for public health research (20 ng/mL)^36^, the country presents three differentiated realities that require further study to serve as input for the development of public health policies. For example, considering the Austral zone, there is evidence in Ireland, which has a similar latitude (51º-55º North), that 25(OH)D synthesis in older adults occurs preferentially in the summer months, making it necessary to provide vitamin D through intake during the winter months^32^.

Older adults living in rural areas are less likely to have 25(OH)D deficiency, a finding that is consistent with some precedents^33^ but differs from others^30^. These differences may be due, in part, to the greater pollution in large cities in developing countries^46^, which impedes adequate 25(OH)D synthesis. It has also been observed that people in rural environments tend to have greater solar exposure than people in cities^47^. It should be noted that Santos and collaborators^30^ emphasized that their results are probably due to a confounding variable since the metropolitan area of Lisbon is where more people with higher incomes live, which is a protective factor against 25(OH)D deficiency.

In contrast, Indigenous peoples’ older adults in Latin America have shown lower levels of 25(OH)D compared with the rest of their country’s population^34^. It is possible that, in the case of Chile, the combination of native people’s darker skin pigmentation^40^ and a higher proportion of them living in the central and southern regions of the country (i.e., at a higher southern latitude) may contribute to understanding this finding. In addition, Indigenous populations tend to use clothing to protect themselves from the climate, which reduces sun exposure (e.g., shawls and hats). Clothing that covers more of the body has been observed as a factor associated with lower 25(OH)D levels^45^.

One of the limitations of this work is that the NHS 2016–2017 is the first national survey that addresses the measurement of 25(OH)D levels in some specific groups, so there are no antecedents with which to contrast results. Second, the descriptive variables were taken based on self-reports, and their responses could be biased, such as the time of sun exposure and ethnicity. Third, as indicated, no cut-off point is defined by the Chilean authorities to distinguish between deficient and insufficient levels. The only suggested cut-off point for the categories referred to in this study as “deficiency” and “no deficiency” were used. Also, BMI was not included as an independent variable. Finally, sampling was carried out over several months, passing through different seasons of the year (part of the winter and the entire spring and summer), which could generate some distortion in the measurement.

It is worth noting that this research used information from the Health Surveys for epidemiological surveillance of the Undersecretariat of Public Health. The authors thank the Ministry of Health of Chile for making the database available. All the results obtained from this research are the responsibility of the authors and in no way compromise that institution.

## Conclusions

The social determinants related to deficient 25(OH)D concentrations in older adults in Chile are mainly associated with lower sun exposure related to variables such as having an urban residence, living in the southernmost areas of the country, and reporting low exposure to sunlight; consistently, residing in the northern part of the country, closer to the Equator, is a protective factor. Other variables, such as being female and belonging to native peoples, also have a negative effect. Our results suggest three differentiated zones within the country regarding latitude (and, thus, sun exposure), which require further attention and study to contribute as input for generating public health policies.

## Data Availability

The National Health Survey database is available in MINSAL webpage, http://epi.minsal.cl/encuesta-nacional-de-salud-2015-2016/.
